# Maternal depression over 7 years postpartum: trajectories and multidimensional predictors in a longitudinal cohort study

**DOI:** 10.4069/whn.2025.11.24

**Published:** 2025-12-31

**Authors:** JaeEun Yang, Ah Rim Kim

**Affiliations:** 1 Department of Health Technology R&D, Bureau of Health R&D and Innovation, Korea Health Industry Development Institute, Korea; 2 Department of Nursing, College of Healthcare Science, Far East University, Korea

**Keywords:** Child development, Depression, Longitudinal studies, Maternal health, Postpartum period

## Abstract

**Purpose:**

Maternal depression can persist beyond the immediate postpartum period and can adversely affect maternal functioning and child development. However, evidence describing long-term trajectories and multidimensional predictors remains limited. This study investigated the 7-year trajectory of maternal depressive symptoms from childbirth to when the child reached 6 years of age and identified significant predictors across child, maternal, and family domains.

**Methods:**

In total, 1,030 mothers from the Panel Study on Korean Children were included. Depressive symptoms were assessed at postpartum years 1, 3, 5, and 7 using the Kessler Depression Scale. Latent growth modeling was used to examine symptom trajectories, and logistic regression analyses were conducted to identify predictors of depressive-symptom risk.

**Results:**

Overall, 55.6% of mothers experienced at least one episode of mild-to-moderate or severe depressive symptoms. Among at-risk mothers, depressive symptoms demonstrated a slight upward trajectory with significant individual variability (linear model fit: *χ*^2^=12.65, root mean square error of approximation=.05, comparative fit index=.96). Significant predictors included preterm or low-birth-weight delivery (odds ratio [OR]=2.29), prenatal depression (OR=2.61), postpartum depression at 1 month (OR=3.41), high parenting stress (OR=1.63), low self-esteem (OR=2.33), and marital conflict (OR=2.02). Higher educational attainment emerged as a protective factor (OR=0.59).

**Conclusion:**

These findings demonstrate the persistence of maternal depressive symptoms and highlight key predictors for promptly identifying mothers at elevated risk. Integrating routine longitudinal screening with interventions targeting maternal stress, self-esteem, and family functioning may be critical for mitigating long-term depressive trajectories. Family-centered approaches, including partner involvement and conflict-management strategies, appear essential for improving maternal mental health and promoting healthier developmental environments for children.

## Introduction

Maternal mental health during the postpartum period is a critical public health concern, with profound implications for both mothers and their children. The transition to motherhood involves substantial physical, psychological, and emotional changes, rendering women particularly vulnerable to mood disorders. Postpartum depression (PPD), characterized by persistent sadness, fatigue, and impaired functioning, represents a serious condition that is distinct from transient “baby blues” or rarer conditions such as postpartum psychosis. According to the U.S. Centers for Disease Control and Prevention, approximately 13% of mothers in the United States experience PPD, with reported prevalence rates ranging from 6.5% to 23.5% depending on geographic and sociodemographic factors [1]. Similarly, studies in Korea indicate that 10% of mothers experience mild-to-moderate PPD, while 3% experience severe depression at 4 weeks postpartum [2].

PPD has far-reaching consequences not only for maternal well-being but also for child development. Evidence suggests that untreated maternal depression adversely affects parenting practices, increases parenting stress, and disrupts mother–infant bonding, ultimately contributing to poorer emotional and cognitive outcomes in children [3,4]. Despite its clinical and public health significance, the long-term trajectory of maternal depression remains insufficiently examined, particularly in studies that simultaneously consider child, maternal, and family factors. Prior research has documented heterogeneous patterns of maternal depression, including linear increases, stabilization, and fluctuating trajectories, shaped by psychological, social, and environmental predictors [5,6].

The postpartum period represents not only a vulnerable phase for maternal mental health but also a foundational stage for child development and family functioning. When maternal depression remains untreated, it can disrupt early attachment processes, generating cascading effects that compromise children’s emotional regulation, cognitive development, and social functioning. Woolhouse et al. [7] reported that approximately one-third of Australian mothers experienced depressive symptoms during the first 4 years postpartum, underscoring both the prevalence and persistence of maternal depression. Moreover, depressive symptoms have been shown to persist or even worsen for up to 21 years postpartum in some mothers, highlighting the need for long-term monitoring and intervention strategies [8].

In Korea, longitudinal research examining maternal depression remains limited, as most studies have focused on single time points or relatively short postpartum periods. Nevertheless, Lee and Kim [9] found that 27% of mothers reported clinically significant depression when their children reached 5 years of age, suggesting a potential increase in depressive symptoms as children grow older. These findings are consistent with international evidence and underscore the importance of extended follow-up studies to capture the evolving nature of maternal depression across cultural and familial contexts.

Maternal depression does not occur in isolation but rather reflects a complex interplay of multiple determinants. Child-related factors, such as preterm birth, low birth weight, and developmental delays, are consistently associated with elevated maternal depression risk [10]. Maternal characteristics, including lower educational attainment, heightened prenatal and PPD symptoms, elevated parenting stress, and low self-esteem, have also been identified as significant predictors [11]. In addition, family dynamics, particularly perceived marital conflict, further amplify risk, underscoring the importance of relational and environmental contexts in shaping maternal mental health [8].

Despite these advances, substantial gaps remain in understanding how child, maternal, and family factors jointly influence maternal depression trajectories over time. Informed by Mercer’s Becoming a Mother framework [12,13] and Bronfenbrenner’s ecological model [14], this study examined maternal depressive symptoms within an integrated, multilevel context. Predictors were classified into child, maternal, and family domains to reflect individual resources, infant-related factors, and proximal family dynamics [12-14]. By identifying early predictors and risk factors, this research aims to inform targeted interventions that support maternal mental health and foster healthier family environments over the long term.

## Methods

**Ethics statement:** This study was approved by the Institutional Review Board of Far East University (No. FEUIRB-20250317-01-04) and was exempted from review because it involved secondary data analysis of publicly available national data that were fully anonymized.

### Study design

This study aimed to address these gaps by examining longitudinal data from a panel study of Korean children, with a focus on maternal depression trajectories from the prenatal period through 7 years postpartum.

This study utilized data from the Panel Study on Korean Children (PSKC) conducted by the Korea Institute of Child Care and Education [15]. Reporting followed the STROBE guidelines (https://www.strobe-statement.org/). The analysis covered data collected from the first year (2008) to the seventh year (2014).

### Participants

The PSKC sample was derived using a stratified multistage sampling method. In the first stage, among medical institutions nationwide, 30 medical facilities were selected as sample institutions. These facilities were chosen through systematic sampling from institutions that had recorded at least 500 childbirths in 2006 across six regions in Korea. In the second stage, from April to July 2008, 2,562 pregnant women who were willing to participate were recruited as preliminary subjects from the sampled institutions. In the third stage, 2,150 newborn households that completed the survey were finalized as panel participants.

**Figure 1.**
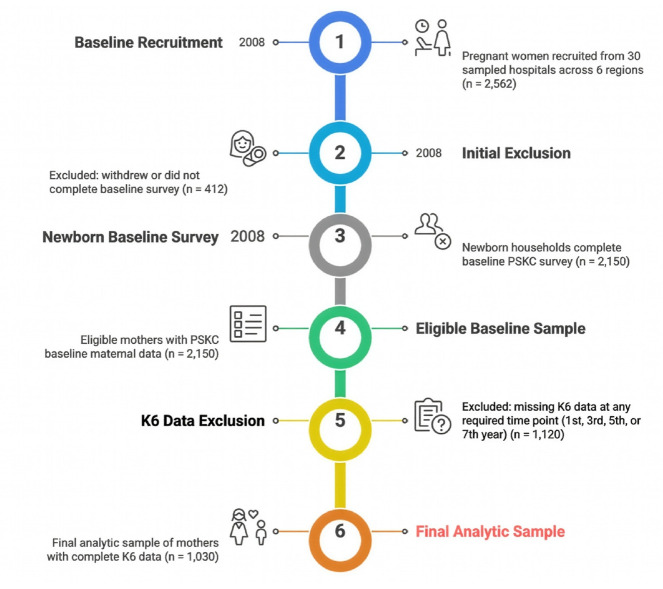
Flow diagram of participant selection for the final analytic sample.

To examine changes in maternal depression trajectories and their predictive factors before and after childbirth, cases with missing responses on the Kessler Depression Scale (K6) administered at the first year (age 0 year), third year (age 2 years), fifth year (age 4 years), and seventh year (age 6 years) were excluded without imputation. This resulted in a final analytic dataset of 1,030 cases. Rather than imputing missing values, the study applied an available-case (listwise deletion) approach, analyzing only cases with valid responses. Consequently, the total number of responses for some categorical variables does not always sum to 1,030. The selection process for the final analytic sample is illustrated in Figure 1.

### Conceptual framework and rationale for variable grouping

This study was theoretically grounded in Mercer’s maternal role attainment (Becoming a Mother) framework and Bronfenbrenner’s ecological systems theory. Mercer’s framework conceptualizes maternal adaptation as a developmental and interactive process shaped by personal resources (e.g., self-esteem), role-related demands (e.g., parenting stress), and proximal relationships (e.g., partner or marital context) [12,13]. Bronfenbrenner’s model situates maternal mental health within nested contexts that span the individual and child level, the family microsystem, and broader environments [14]. Consistent with these perspectives, and with Belsky’s process of parenting model in early childhood, which organizes determinants into parental resources, child characteristics, and contextual stress or support [16,17], predictors were grouped into three domains: child characteristics (e.g., preterm or low birth weight, neonatal intensive care unit [NICU] admission, early developmental risk), maternal characteristics (e.g., education, prenatal and PPD symptoms, parenting stress, self-esteem, and obstetric or behavioral factors), and family characteristics (e.g., marital conflict, paternal involvement, socioeconomic status, and the couple’s reproductive context, such as planned pregnancy and assisted reproduction). Within this framework, planned pregnancy and the use of assisted reproductive technology (ART) were classified as family-level variables because they reflect joint reproductive decision-making and the shared reproductive context of the couple rather than a solely individual maternal attribute. Couple-level research has demonstrated that agreement or disagreement in pregnancy intentions [18,19] and decisions related to ART [20] predict fertility and family outcomes more accurately when both partners’ perspectives are considered than when only one partner is assessed [18-20].

### Measurement

#### Outcome variable: depression

Maternal mental health, specifically depression risk status, was classified using the Korean version of the Kessler Depression Scale [15]. The K6 assesses six items related to depressive symptoms, including anxiety, lethargy, and restlessness, using a 5-point Likert scale ranging from “never” (1 point) to “always” (5 points) over the past 30 days. As a standardized instrument originally developed for the U.S. National Health Interview Survey, the K6 distinguishes between individuals with and without mental disorders. Following the scoring system proposed by Kessler et al. [20], item responses were recoded on a scale from 0 to 4, yielding a total possible score ranging from 0 to 24. Scores were categorized as 0–7 (normal), 8–12 (mild-to-moderate depression), and 13–24 (severe depression). Consistent with prior research by Lee and Kim [9] using PSKC data, mothers who scored in the “mild-to-moderate depression” or “severe depression” range at any assessment point in the first, third, fifth, or seventh year were classified into the depression risk group. The internal consistency of the K6 scale in this study was high, with Cronbach’s alpha values of .90, .91, .92, and .92 at the first, third, fifth, and seventh years, respectively. In this study, K6 scores were used to indicate levels of maternal depressive symptoms and to classify mothers into depression risk groups; they were not used as a clinical diagnosis of PPD.

#### Predictor variables

Guided by Mercer’s maternal role attainment framework and Bronfenbrenner’s ecological systems theory, and consistent with Belsky’s process of parenting model in early childhood, predictors were organized into child, maternal, and family characteristics [12-14,16,17].

##### 1) Child characteristics

*Preterm or low birth weight:* Preterm infants (gestational age <37 weeks) or low birth weight infants (<2,500 g) were coded as 1, whereas full-term infants with normal birth weight were coded as 0.

*NICU admission:* Neonates who were admitted to the NICU after birth were coded as 1, and those who were not admitted were coded as 0.

*Developmental risk:* Developmental risk was classified based on results from the Korean Denver II Developmental Screening Test [21]. The Denver II, adapted for Korean populations in 2002, assesses four developmental domains: personal–social, fine motor–adaptive, language, and gross motor skills. Infants who scored as “delay” on at least one item or as “caution” on two or more items were categorized as “developmentally at risk.” For this study, children assessed as “at risk” at least once before 3 years of age (first to third years) were coded as 1, whereas all others were coded as 0.

##### 2) Maternal characteristics

*Advanced maternal age:* Mothers aged ≥35 years at the time of childbirth were coded as 1, whereas those younger than 35 years were coded as 0.

*Educational attainment:* High school or below was coded as (1), vocational/technical college as (2), bachelor’s degree as (3), and postgraduate as (4).

*Smoking:* Mothers who smoked regularly or who temporarily stopped smoking due to pregnancy or breastfeeding were coded as 1, whereas mothers who did not smoke were coded as 0.

*Primiparous mothers:* Mothers giving birth to their first child were coded as 1, and multiparous mothers were coded as 0.

*Delivery mode:* Vaginal delivery was coded as (1), planned cesarean section as (2), and emergency cesarean section as (3), with vaginal delivery as the reference.

*Employment or schooling status:* Mothers who were employed or attending school were coded as 1, whereas those who were unemployed or not attending school were coded as 0. This dichotomization reflects a well-established distinction in mental health research between active social-role engagement and social disengagement, the latter of which has been consistently associated with an increased risk of depressive symptoms in postpartum women.

*Prenatal/postpartum depression:* Prenatal and PPD symptoms were assessed using the Kessler Depression Scale (K6) [22], which was administered multiple times around childbirth. For the present study, two time points corresponding to variables available in the analytic dataset were used: (1) prenatal depressive symptoms assessed one month before delivery (T1) and (2) PPD symptoms assessed one month after delivery via telephone survey (T2). Both assessments reflected symptoms experienced during the preceding 30 days, consistent with the K6 response framework. Using the same scoring approach as for the outcome variable, all K6 responses were recoded on a 0–24 scale. Mothers scoring ≥8 points were coded as 1 (depressed), whereas those scoring <8 points were coded as 0 (normal). The internal consistency (Cronbach’s alpha) for the prenatal and postpartum K6 assessments in this study was .82 and .81, respectively.

*Parenting stress:* Perceived parenting stress was assessed using 10 items from Kim and Kang’s Parenting Stress Scale [22]. Items were rated on a 5-point Likert scale ranging from “not at all” (1 point) to “very much” (5 points), yielding total scores ranging from 10 to 50, with higher scores indicating greater parenting stress. Participants scoring above the sample mean were classified as the stress risk group (1), whereas those scoring at or below the mean were categorized as normal (0). The reliability of this scale in the present study was shown by a Cronbach’s alpha value of .84.

*Self-esteem:* Self-esteem was measured using the Korean version [21] of Rosenberg’s 10-item Self-Esteem Scale [23]. Items were rated on a 4-point Likert scale (1 strongly disagree to 4 strongly agree), and negatively worded items were reverse-coded. Total scores ranged from 10 to 40, with higher scores indicating greater self-esteem. Participants scoring below the sample mean were classified as the low self-esteem group (1), whereas those scoring at or above the mean were categorized as normal (0). The reliability of this measure in the present study was shown by a Cronbach’s alpha value of .85.

##### 3) Family characteristics

Family characteristics captured the proximal relational and socioeconomic environment of the household, as well as the couple-level reproductive context. In the PSKC, the planned pregnancy item indicates whether the pregnancy was planned and desired by both parents, and the ART variable reflects whether the couple used fertility treatments such as ovulation induction, artificial insemination, or in vitro fertilization. Although these variables were reported by mothers, they are conceptually grounded in dyadic, couple-level decision-making and were therefore classified as family-level contextual characteristics within the microsystem.

*Basic livelihood status:* Families receiving basic livelihood benefits or classified as low-income households were coded as 1, whereas all other families were coded as 0.

*Planned pregnancy:* Pregnancies that were planned and desired by both parents were coded as 1, whereas all other pregnancies were coded as 0.

*ART:* Conceptions achieved using fertility treatments, such as ovulation induction or in vitro fertilization, were coded as 1, whereas natural conceptions were coded as 0.

*Marital conflict:* Marital conflict was assessed using an 8-item modified scale developed by Jeong [24]. Items were rated on a 5-point Likert scale (1 not at all to 5 very true), with total scores ranging from 8 to 40, and higher scores indicating greater levels of marital conflict. Participants scoring above the sample mean were classified as the conflict group (1), whereas those scoring at or below the mean were categorized as normal (0). The reliability of this scale in the present study was .90.

*Paternal involvement:* Paternal involvement in childcare was assessed using a modified version of an established scale [25]. The scale consisted of four items, such as “The father feeds or bathes the baby,” rated on a 5-point Likert scale (1 not at all to 5 very true). Total scores ranged from 4 to 20, with higher scores indicating greater paternal involvement. Participants scoring below the sample mean were classified as the low involvement group (1), whereas those scoring at or above the mean were categorized as normal (0). The reliability of this scale in the present study was .75.

Finally, for this study, parenting stress, marital conflict, self-esteem, and paternal involvement were dichotomized using sample mean cutoff scores. Participants scoring above the sample mean on each scale were classified as the risk group (1), whereas those scoring at or below the mean were classified as the normal group (0). This approach has been commonly used in previous studies utilizing these instruments.

### Data analysis

Data were analyzed using IBM SPSS ver. 23.0 and AMOS ver. 23.0 (IBM Corp., Armonk, NY, USA). Nominal variables were summarized using frequencies and percentages, whereas continuous variables were described using means and standard deviations. These descriptive statistics provided an overview of participant characteristics and the distribution of predictor variables.

To examine changes in maternal depressive symptoms from the prenatal period (T1) through the seventh year postpartum (T6), latent growth model (LGM) analyses were conducted for the subgroup classified as at risk. Two unconditional models, a no-change model and a linear change model, were compared using multiple fit indices (*χ*^2^/df, root mean square error of approximation [RMSEA], Tucker-Lewis Index [TLI], comparative fit index [CFI], and normed fit index [NFI]) to identify the trajectory that best represented changes in depressive symptoms over time.

To estimate changes in depression levels over 7 years within the at-risk group, an LGM analysis was conducted to examine symptom trajectories across time. The normal group (N=457), whose depression scores remained within the normal range throughout the 7-year follow-up, was excluded from this analysis because their symptom change patterns were not informative for trajectory modeling. Based on mean depression levels observed in the first, third, fifth, and seventh years (Table 1), two models were specified: a no-change model, assuming no variation across time points, and a linear change model, assuming consistent change between time points. Model fit was evaluated using normed *χ*^2^ (*χ*^2^/df ≤3), RMSEA (≤.05), and incremental fit indices, including the TLI (≥.90), CFI (≥.90), and NFI (≥.90).

Fit indices indicated that the linear change model (*χ*^2^=12.65, df=5, *p*=.03, TLI=.95, CFI=.96, NFI=.94, RMSEA=.05) provided a significantly better fit than the no-change model (*χ*^2^=37.26, df=5, *p*<.001, TLI=.80, CFI=.83, NFI=.81, RMSEA=.11). The linear change model demonstrated lower *χ*^2^ values, acceptable *χ*^2^/df ratios (<3), and superior fit indices, with CFI and NFI exceeding recommended thresholds. These findings indicate that depressive symptom levels in the at-risk group changed consistently over time. Accordingly, the linear change model was selected to provide a clearer representation of the depression trajectory in this group (Supplementary Table 1).

Logistic regression analyses using the enter method were conducted to identify predictors associated with maternal depressive symptom risk during the 7-year postpartum period. Predictor variables included child characteristics (e.g., preterm or low birth weight, NICU admission, developmental risk), maternal characteristics (e.g., educational attainment, prenatal and PPD symptoms, parenting stress, self-esteem, parity, smoking, mode of delivery, and employment or schooling status), and family characteristics (e.g., marital conflict, paternal involvement, socioeconomic status, planned pregnancy, and ART). Odds ratios (ORs) and 95% confidence intervals (CIs) were calculated to estimate the magnitude of associations.

## Results

### Characteristics of the participants

The characteristics of the participants are presented in Table 2. Among child-related characteristics, male children accounted for 51.4% of the sample, with a mean gestational age of 39.22 weeks (SD=1.19) and a mean birth weight of 3.26 kg (SD=0.42). The majority of children (94.8%) were full-term infants with normal birth weight, whereas 4.0% had received treatment in the NICU. The mean ages of children in the first (T3), third (T4), fifth (T5), and seventh (T6) years were 5.33 months (SD=0.97), 26.27 months (SD=1.32), 51.13 months (SD=1.18), and 75.19 months (SD=1.43), respectively. Developmentally at-risk children, defined as those with at least one “suspected delay” on the Denver Developmental Screening Test during the first to third years, accounted for 35.0% of the sample.

Among maternal characteristics, the mean maternal age in the first year was 31.3 years (SD=3.7), whereas the mean paternal age was 33.8 years (SD=4.0). Mothers aged 35 years or older comprised 18.4% of the sample. Educational attainment was distributed as follows: college or higher (37.9%), vocational or technical college (31.5%), and high school or below (30.2%). Unemployment or non-enrollment in education during the childbirth year was observed in 72.3% of mothers, while 90.9% were neither recipients of basic living subsidies nor classified as low-income households. Multiparous mothers (55.2%) outnumbered primiparous mothers (44.7%). Modes of delivery included vaginal delivery (55.2%), planned cesarean section (27.1%), and emergency cesarean section (17.7%). The majority of mothers (95.5%) conceived naturally, with only 4.3% using fertility treatments such as ovulation induction, artificial insemination, or in vitro fertilization. Immediate contact between mothers and newborns after birth was reported in 68.6% of cases. In addition, 71.8% of pregnancies were planned and desired by both parents, whereas more than half of mothers reported no intention to have additional children (50.5%) or expressed uncertainty (22.0%), indicating a relatively negative outlook toward subsequent childbirth.

### Changes in prenatal and postpartum depression levels and classification of maternal depression across 7 years

Depression levels among 1,030 women who gave birth in 2008 were assessed at the prenatal period (1 month before delivery, T1), early postpartum (1 month after delivery, T2), and subsequently at two-year intervals (first year, T3; third year, T4; fifth year, T5; seventh year, T6), as presented in Table 1. The mean depression scores were 5.47 at the prenatal assessment (T1), 3.11 at 1 month postpartum (T2), 5.54 at the first year postpartum (T3), 5.81 (SD=4.26) at the third year (T4), 5.78 (SD=4.52) at the fifth year (T5), and 5.61 (SD=4.50) at the seventh year (T6). These results indicate that the lowest mean depression score occurred at 1 month postpartum (T2), followed by stabilization in the mid–5-point range over the subsequent 7 years, with the highest mean observed at the third year (T4), when children were approximately 2 years old.

Clinically depressed mothers, classified as experiencing mild-to-moderate or severe depression, accounted for 26.9%–29.3% of the sample at all time points except the early postpartum period, when the proportion was 12.1%. Overall, the depression risk group comprised 55.6% of the sample, whereas the normal group accounted for 44.4%.

For the normal group, mean depression scores across the six time points were 4.20 (SD=3.14) at T1, 2.08 (SD=0.79) at T2, 3.00 (SD=2.54) at T3, 3.15 (SD=2.65) at T4, 3.03 (SD=2.55) at T5, and 2.89 (SD=2.50) at T6. In contrast, mean depression scores in the depression risk group were 6.49 (SD=4.27) at T1, 3.93 (SD=3.99) at T2, 7.56 (SD=4.11) at T3, 7.93 (SD=4.12) at T4, 7.98 (SD=4.54) at T5, and 7.78 (SD=4.56) at T6. This group demonstrated a decrease in depression scores at T2, followed by a peak at T5 (fifth year), with overall averages indicating clinically significant mild-to-moderate depression levels from the first through the seventh year.

In addition, the number of time points at which mothers experienced mild-to-moderate or severe depression was distributed as follows: 23.1% experienced depression at one time point, 14.4% at two time points, 10.0% at three time points, and 8.2% at all assessed time points during the 7-year follow-up period (Table 3).

### Descriptive statistics of predictor variables

Maternal parenting stress was slightly below the mid-range level, with a mean score of 27.59 (SD=6.15). Maternal self-esteem was moderately above the mid-range level, with a mean score of 30.02 (SD=4.12), whereas marital conflict was relatively low, with a mean score of 15.95 (SD=6.02) (Table 2).

### Trajectory of postpartum depression changes in the at-risk group

Estimated depression values for the at-risk group, analyzed using the selected linear change model and the no-change model, are presented in Table 4. In the linear change model, the mean and variance of the initial level (intercept) were 7.70 (*p*<.001) and 5.11 (*p*<.001), respectively, indicating statistically significant individual differences in maternal depression levels at the first year (T3). The mean rate of change (slope) was 0.07, reflecting a slight increase in depression levels over the 7-year postpartum period; however, this increase was not statistically significant (*p*=.382). The variance of the rate of change was 1.12 (*p*<.001), demonstrating significant individual differences in depression change trajectories over time.

The covariance between the initial level and the rate of change was −0.86 (*p*>.05), indicating no statistically significant association between maternal depression levels at the first year (T3) and subsequent changes in depression as children aged to 6 years. These findings suggest that although initial depression levels differed substantially across mothers, patterns of change over time were heterogeneous rather than uniform.

### Exploration of predictive factors for maternal depressive symptoms until the child reaches age 6

The findings of the logistic regression analysis using the enter method are presented in Table 5.

#### Child characteristics

Mothers of preterm or low-birth-weight infants (OR=2.29, 95% CI=1.01–5.20, *p*=.047) were approximately 2.3 times more likely to experience depressive symptoms than mothers of full-term, normal-weight infants.

#### Maternal characteristics

Final educational attainment at the bachelor’s degree level or higher (OR=0.59, 95% CI=0.39–0.90, *p*=.014) was associated with a 41% lower likelihood of maternal depression compared with high school education or below. Mothers classified in the prenatal depression risk group (OR=2.61, 95% CI=1.73–3.94, *p*<.001) and PPD risk group (OR=3.41, 95% CI=1.92–6.05, *p*<.001) were 2.6 and 3.4 times more likely, respectively, to experience depressive symptoms over the 7-year period. Higher parenting stress (OR=1.63, 95% CI=1.15–2.32, *p*=.006) increased the likelihood of maternal depression by approximately 1.6 times, while low self-esteem (OR=2.33, 95% CI=1.63–3.31, *p*<.001) was associated with more than a twofold increase in risk.

#### Family characteristics

Maternal perception of marital conflict (OR=2.02, 95% CI=1.43–2.87, *p*<.001) was significantly associated with a twofold increase in the likelihood of experiencing depressive symptoms.

## Discussion

This study contributes to the expanding body of research on maternal PPD symptoms by examining longitudinal trajectories over 7 years following childbirth and by identifying multidimensional predictors that influence these trajectories. The findings reveal an intricate interplay among maternal, child, and family factors, demonstrating both alignment with and divergence from previous research. Our domain-specific findings are consistent with Mercer’s proposition that maternal adaptation is shaped by personal resources and role-related stress, as well as with Bronfenbrenner’s view that proximal family processes, such as marital interactions and paternal involvement, are critical determinants of maternal mental health trajectories [12–14]. The three-domain grouping applied in this study also mirrors Belsky’s process model, which conceptualizes influences across parent, child, and contextual domains, thereby supporting the interpretability and external validity of the results [16].

A striking finding of this study is the high prevalence of clinically significant depressive symptoms, observed in 55.6% of mothers across the 7-year postpartum period, which markedly exceeds the 13.6% prevalence reported in a multinational study spanning six countries [26]. Because the K6 is a brief and sensitive screening instrument, these estimates reflect elevated depressive symptom risk rather than the prevalence of clinically diagnosed PPD. The K6 has demonstrated strong criterion validity and high sensitivity for identifying clinically significant psychological distress in population-based studies [27], which may partially explain the higher risk estimates compared with postpartum-specific instruments such as the Edinburgh Postnatal Depression Scale or the Patient Health Questionnaire-9. These discrepancies underscore the potential influence of cultural, socioeconomic, and methodological differences, including variation in follow-up duration and measurement tools.

Educational attainment emerged as a consistent predictor of maternal depressive symptoms, echoing findings by Tsai et al. [28], who reported higher odds of clinically diagnosed PPD among mothers with a high school education or less. The present study reinforces the association between lower educational attainment and sustained depression risk, highlighting the importance of targeted interventions aimed at mitigating educational disparities. Child-related factors, such as preterm birth and low birth weight, also played a pivotal role, aligning with prior literature that identifies neonatal health challenges as significant stressors for maternal mental health. Low Apgar scores and neonatal illnesses have been consistently associated with an increased risk of postpartum depressive symptoms in previous studies [29,30]. The longitudinal design of this study further underscores the persistent influence of these child-related factors, extending well beyond the immediate postpartum period and underscoring the need for sustained support for affected mothers and infants.

Accumulating evidence suggests that support for mothers at risk of persistent depressive symptoms should be conceptualized as a sustained, multicomponent care approach. Psychological and parenting-focused interventions have been shown to yield small-to-moderate reductions in perinatal depressive symptoms and, in some cases, to improve infant outcomes [31,32]. Social support and partner-inclusive interventions further indicate that enhancing perceived support [33], dyadic communication [34], and shared coping strategies [35] may alleviate maternal distress, although more rigorous trials remain necessary [33,34]. In addition, community-based and home-visiting interventions appear particularly beneficial for vulnerable mothers by providing ongoing contact, case management, and tailored psychosocial support [29,35]. Collectively, these findings suggest that longitudinal screening efforts should be explicitly linked to accessible psychosocial and family-oriented services to sustain improvements in maternal mental health.

The influence of family dynamics, particularly marital conflict, represents a pivotal factor in shaping maternal mental health outcomes. This study corroborates findings from prior research conducted among Korean mothers [36], which consistently demonstrate that heightened marital discord is strongly associated with increased depressive symptoms. Such conflict not only intensifies maternal distress but may also spill over into parenting practices and child behavior. These findings align with evidence from a scoping review synthesizing international studies [37], which showed that family-inclusive interventions can effectively reduce PPD symptoms, underscoring the central role of familial support networks in mitigating stress and facilitating recovery.

Beyond maternal outcomes, marital conflict and maternal depression have well-documented implications for child development. Maternal depression has been associated with delayed child development, partly due to reduced responsive caregiving [38], with similar findings reported in studies showing diminished developmental functioning by age three among children of depressed mothers [39]. These associations highlight the importance of addressing both maternal and familial factors in intervention strategies, as chronic maternal depression has also been linked to impairments in children’s executive functioning, particularly in the context of elevated household stress [40].

This study reinforces the importance of early, comprehensive interventions that integrate maternal mental health support with family-centered approaches. Programs aimed at reducing marital conflict and strengthening supportive family environments may substantially mitigate the adverse effects of maternal depression on both mothers and children. Future research should continue to employ longitudinal designs to further elucidate the dynamic interplay among family functioning, maternal mental health, and child development, thereby informing the development of tailored interventions that address these interconnected challenges.

The pattern of predictors identified in this study is consistent with multilevel models of maternal adjustment. Lower educational attainment emerged as a robust predictor of depressive symptoms, aligning with prior evidence that mothers with lower educational levels often encounter greater barriers to mental health resources, experience higher parenting stress, and face increased challenges in negotiating parenting and household responsibilities [5,8]. Recent reviews further suggest that limited educational resources may constrain access to effective coping strategies and communication skills, thereby exacerbating stress and relationship strain during the postpartum period [31,33]. Educational attainment is therefore likely to interact with both parenting stress and marital dynamics, amplifying risk when coping and communication capacities are constrained.

Elevated prenatal and PPD symptoms were strong predictors of persistent symptom trajectories across the 7-year follow-up period, consistent with prior longitudinal studies demonstrating that early perinatal distress often persists in the absence of sustained support and serves as a key determinant of long-term maternal mental health trajectories [7,8]. These findings underscore the importance of early identification and timely intervention for women exhibiting elevated depressive symptoms during pregnancy and the early postpartum period.

Parenting stress and low maternal self-esteem also showed strong associations with depressive symptoms. Meta-analytic evidence indicates that interventions targeting parenting confidence, stress management, and maternal efficacy yield meaningful reductions in perinatal depressive symptoms, particularly when tailored to mothers experiencing high levels of stress [31,32]. These findings support the view that mothers with lower confidence in their parenting role may be especially vulnerable to chronic depressive trajectories.

Finally, marital conflict demonstrated one of the strongest effects among family-level predictors. This finding is consistent with prior literature showing that low partner support [33], poor dyadic coping [34], and communication breakdowns [34] substantially heighten maternal vulnerability to depression. Reviews of partner- and family-inclusive interventions have reported improvements in perceived social support [33], dyadic communication [34], and maternal mood when partners are actively involved in psychoeducational or supportive programs [29]. These findings reinforce the importance of couple- and family-centered approaches as integral components of long-term maternal mental health care.

This study has several limitations. First, as a secondary data analysis of the PSKC, the variables were limited to those available in the original dataset, and potentially important predictors of maternal depression could not be examined. Second, all measures relied on maternal self-report, which may introduce reporting or recall bias. Third, depressive symptoms were assessed using the K6, a screening tool rather than a diagnostic instrument, and therefore the prevalence estimates reported here may not correspond directly to clinically diagnosed depressive disorders. Finally, although the data were longitudinal, causal inferences cannot be drawn. Future studies should incorporate diagnostic assessments and additional psychosocial, biological, and contextual variables to develop a more comprehensive understanding of long-term maternal depression trajectories. Additionally, maternal employment and schooling were dichotomized to reflect the broader distinction between active role engagement and social disengagement, which has been strongly associated with PPD symptoms in prior psychosocial research. Nevertheless, employment and schooling may involve qualitatively distinct forms of social participation, role demands, and stress exposure. Future studies using more nuanced classifications or dyadic work–family interaction measures would help clarify these differential pathways more precisely.

In conclusion, this study highlights the complexity and persistence of maternal depression during the first 7 years postpartum and identifies significant predictors across child, maternal, and family domains. Although more than half of the mothers experienced clinically significant depressive symptoms at least once during this period, variability in initial symptom levels and longitudinal trajectories underscores the individualized nature of maternal depression. Key predictors included preterm or low-birth-weight delivery, lower maternal educational attainment, elevated prenatal and PPD symptoms, high parenting stress, low self-esteem, and perceived marital conflict, emphasizing the need for comprehensive, family-centered interventions. Screening approaches that incorporate these predictors as early indicators could substantially enhance the identification of high-risk mothers, facilitating timely and targeted intervention efforts. By addressing these multidimensional factors early, healthcare providers may mitigate long-term adverse consequences for both mothers and children, thereby promoting improved developmental outcomes and overall family well-being. Further research should examine these dynamics across diverse populations and extended time frames to inform the development of more effective prevention and intervention strategies.

## Figures and Tables

**Table 1.** Changes in depression levels over 7 years postpartum (N=1,030)

Variable	Categories	n (%) or mean±SD					
		Prenatal 1 month (T1)	Postpartum 1 month (T2)	First year (T3)	Third year (T4)	Fifth year (T5)	Seventh year (T6)
Depression level	Normal (<8)	753 (73.1)	905 (87.9)	737 (71.6)	728 (70.7)	735 (71.4)	741 (71.9)
	Mild-moderate (8–12)	223 (21.7)	103 (10.0)	235 (22.8)	234 (22.7)	218 (21.2)	218 (21.2)
	Severe (13–24)	54 (5.2)	22 (2.1)	58 (5.6)	68 (6.6)	77 (7.5)	71 (6.9)
Changes in depression levels	Overall (n=1,030)	5.47±3.97	3.11±3.62	5.54±4.17	5.81±4.26	5.78±4.52	5.61±4.50
	Normal (n=457)	4.20±3.14	2.08±2.79	3.00±2.54	3.15±2.65	3.03±2.55	2.89±2.50
	At-Risk (n=573)	6.49±4.27	3.93±3.99	7.56±4.11	7.93±4.12	7.98±4.54	7.78±4.56

**Table 2.** General characteristics of study participants and descriptive statistics of predictive variables (N=1,030)

Variable	Categories	n (%) or mean±SD	Range
Child characteristics			
Sex	Male	529 (51.4)	
	Female	501 (48.6)	
Gestational age (week)		39.22±1.19	31.4–41.7
Birth weight (kg)		3.26±0.42	1.2–4.9
Preterm infant or low birth weight infant	Yes	54 (5.2)	
	No	976 (94.8)	
Neonatal intensive care unit admission	Yes	41 (4.0)	
	No	985 (95.6)	
Mean age (month)	First year (2008)	5.33±0.97	4–10
	third year (2010)	26.27±1.32	23–32
	Fifth year (2012)	51.13±1.18	48–54
	Seventh year (2014)	75.19±1.43	72–79
Denver II results	Normal development	485 (47.1)	
	Developmentally at-risk	360 (35.0)	
Maternal characteristics			
Maternal age (year)		31.28±3.69	19–46
Paternal age (year)		33.78±3.91	19–50
Advanced maternal age (≥35 years)	Yes	190 (18.4)	
	No	840 (81.6)	
Final educational attainment	High school or below	311 (30.2)	
	Vocational/technical college	324 (31.5)	
	Bachelor’s degree or higher	390 (37.9)	
Current smoking	Yes	26 (2.5)	
	No	1,004 (97.5)	
Employment or schooling	Yes	285 (27.7)	
	No	745 (72.3)	
Primiparous mother	Yes	460 (44.7)	
	No	569 (55.2)	
Delivery mode	Vaginal delivery	569 (55.2)	
	Planned cesarean	279 (27.1)	
	Emergency cesarean	182 (17.7)	
Postpartum skin contact opportunity with baby	Yes	707 (68.6)	
	No	321 (31.2)	
Future childbirth intention	Yes	282 (27.4)	
	No	520 (50.5)	
	Unsure	227 (22.0)	
Maternal parenting stress (score)		27.59±6.15	1–47
Maternal self-esteem (score)		30.02±4.12	15–40
Family characteristics			
Basic livelihood support	None	936 (90.9)	
	Recipient	12 (1.2)	
	Near-poverty	23 (2.2)	
Planned pregnancy	Yes	740 (71.8)	
	No	289 (28.1)	
Assisted reproduction	Yes	44 (4.3)	
	No (natural)	1028 (95.5)	
Marital conflict (score)		15.95±6.02	8–38
Paternal involvement (score)		14.25±3.16	4–20

Total numbers may not equal 1,030 due to missing data.

**Table 3.** Frequency analysis of maternal depression levels across 7 years (N=1,030)

Maternal depression levels	Number of depressive episodes (time points)	Group classification	n (%)
Mild-to-moderate or severe depression	0	Normal	457 (44.4)
	1	At-risk group	238 (23.1)
	2		148 (14.4)
	3		103 (10.0)
	4		84 (8.2)

**Table 4.** Linear change model and no-change model for depression in the at-risk group (N=573)

Statistic	Initial level	Rate of change	Initial level–rate of change
Mean	7.70^***^	0.07	-
Variance	5.10^***^	1.12^***^	-
Covariance	-	-	-0.86

*** *p*<.001.

**Table 5.** Predictive factors for maternal depression until the child reaches age 6 years

Variable		β	OR (95% CI)	*p*-value
Child characteristics				
Preterm or low birth weight (reference, No)		0.83	2.29 (1.01–5.20)	.047
Neonatal intensive care unit admission (reference, No)		0.18	1.20 (0.52–2.75)	.674
Developmental risk (reference, No)		–0.07	0.94 (0.67–1.30)	.692
Maternal characteristics				
Advanced maternal age (reference, No)		0.07	1.07 (0.70–1.63)	.754
Final educational attainment	High school or below	Reference		.047
	Vocational/technical college	–0.26	0.77 (0.50-1.17)	.222
	Bachelor’s degree or higher	–0.52	0.59 (0.39-0.90)	.014
Current smoking (reference, No)		0.68	1.96 (0.57–6.76)	.285
Primiparous mother (reference, No)		0.23	1.26 (0.87–1.83)	.227
Delivery mode	Vaginal delivery	Reference		.854
	Planned cesarean	0.04	1.04 (0.70-1.56)	.837
	Emergency cesarean	0.13	1.14 (0.72-1.83)	.577
Employment or schooling (reference, No)		0.01	1.01 (0.70–1.83)	.949
Prenatal depression (reference, No)		0.96	2.61 (1.73–3.94)	<.001
Postpartum depression (reference, No)		1.23	3.41 (1.92–6.05)	<.001
Parenting stress (reference, No)		0.49	1.63 (1.15–2.32)	.006
Low self-esteem (reference, No)		0.84	2.33 (1.63–3.31)	<.001
Family characteristics				
Basic livelihood recipient (reference, No)		–0.13	0.88 (0.37–2.05)	.759
Planned pregnancy (reference, Yes)		–0.15	0.86 (0.59–1.25)	.427
Assisted reproductive technology (reference, No)		0.10	1.11 (0.47–2.64)	.815
Marital conflict (reference, No)		0.71	2.02 (1.43–2.87)	<.001
Paternal involvement (reference, No)		0.27	1.31 (0.93–1.84)	.124
Model χ^2^=199.66^***^, Negelkerke R^2^=.30, Hosmer-Lemeshow test, χ^2^=9.18 (*p*=.327).				

OR: Odds ratio; CI: confidence interval; ref: reference category.

*** *p*<.001.

## Supplementary materials

Further details on supplementary materials are presented online (available at https://doi.org/10.4069/whn.2025.11.24).

Supplementary Table 1. Model fit comparison between no-change and linear change models for depression in the at-risk group (N=573)
