# Supplementary material for: Maternal depression over 7 years postpartum: trajectories and multidimensional predictors in a longitudinal cohort study
Source: Womens Health Nurs. 2025 Dec 31;31(4):346–59. doi: 10.4069/whn.2025.11.24 (PMC12835444; doi:10.4069/whn.2025.11.24)
Supplement: Supplementary Table 1. — Model fit comparison between no-change and linear change models for depression in the at-risk group (N=573) [file whn-2025-11-24-Supplementary-Table-1.pdf]

Supplementary Table 1. Model fit comparison between no-change and linear change models for depression in the at-risk group (N=573)

Model	χ^2	df	Normed χ^2	*p*-value	TLI	CFI	NFI	RMSEA
No-change	37.25	5	2.53	<.001	.80	.83	.81	.10
Linear change	12.64	5	7.45	.02	.95	.96	.93	.05

TLI: Tucker-Lewis index; CFI: comparative fit index; NFI: normed fit index; RMSEA: root mean square error of approximation.
